# Intranasal oxytocin versus placebo in the treatment of adults with autism spectrum disorders: a randomized controlled trial

**DOI:** 10.1186/2040-2392-3-16

**Published:** 2012-12-05

**Authors:** Evdokia Anagnostou, Latha Soorya, William Chaplin, Jennifer Bartz, Danielle Halpern, Stacey Wasserman, A Ting Wang, Lauren Pepa, Nadia Tanel, Azadeh Kushki, Eric Hollander

**Affiliations:** 1 Mount Sinai School of Medicine, One Gustave L. Levy Place, New York, NY 10029-6574, USA; 2 Bloorview Research Institute, University of Toronto, 150 Kilgour Road, Toronto, ON M4G 1R8, Canada; 3 St John’s University, 8000 Utopia Parkway, Queens, NY 11439, Jamaica; 4 Child Psychiatry Annex, Albert Einstein College of Medicine and Montefiore Medical Center, 111 E. 210th Street, Bronx, NY 10467-2490, USA; 5 Department of psychiatry, Rush University, 2150 w Harrison st, Chicago, IL, USA

**Keywords:** Autism, Adults, Oxytocin, Clinical trial, Social cognition

## Abstract

**Background:**

There are no effective medications for the treatment of social cognition/function deficits in autism spectrum disorder (ASD), and adult intervention literature in this area is sparse. Emerging data from animal models and genetic association studies as well as early, single-dose intervention studies suggest that the oxytocin system may be a potential therapeutic target for social cognition/function deficits in ASD. The primary aim of this study was to examine the safety/therapeutic effects of intranasal oxytocin versus placebo in adults with ASD, with respect to the two core symptom domains of social cognition/functioning and repetitive behaviors.

**Methods:**

This was a pilot, randomized, double-blind, placebo-controlled, parallel design trial of intranasal oxytocin versus placebo in 19 adults with ASD (16 males; 33.20 ± 13.29 years). Subjects were randomized to 24 IU intranasal oxytocin or placebo in the morning and afternoon for 6 weeks. Measures of social function/cognition (the Diagnostic Analysis of Nonverbal Accuracy) and repetitive behaviors (Repetitive Behavior Scale Revised) were administered. Secondary measures included the Social Responsiveness Scale, Reading-the-Mind-in-the-Eyes Test and the Yale Brown Obsessive Compulsive Scale – compulsion subscale and quality of life (World Health Organization Quality of Life Questionnaire – emotional/social subscales). Full-information maximum-likelihood parameter estimates were obtained and tested using mixed-effects regression analyses.

**Results:**

Although no significant changes were detected in the primary outcome measures after correcting for baseline differences, results suggested improvements after 6 weeks in measures of social cognition (Reading-the-Mind-in-the-Eyes Test, *p* = 0.002, *d* = 1.2), and quality of life (World Health Organization Quality of Life Questionnaire – emotion, *p* = 0.031, *d* = 0.84), both secondary measures. Oxytocin was well tolerated and no serious adverse effects were reported.

**Conclusions:**

This pilot study suggests that there is therapeutic potential to daily administration of intranasal oxytocin in adults with ASD and that larger and longer studies are warranted.

**Trial registration:**

NCT00490802

## Background

Autism spectrum disorder (ASD) is a group of neurodevelopmental disorders characterized by impairments in social functioning, communication and repetitive behaviors. Currently, there are no pharmacologic treatments for social cognition and function deficits in individuals with ASD. Oxytocin and its related pathways have been of particular interest in ASD, because of its unique role in influencing social behaviors and its potential for generating animal models with behavioral deficits that may be relevant to ASD [1]. Emerging genetics data from association studies and few cases with deletions/duplications of the oxytocin receptor (OXT-R) gene suggest that oxytocin may play a role in the pathophysiology of the disorder (for example [2-5]). Additionally, a reduction in oxytocin mRNA has been reported in the temporal cortex of subjects with ASD and associated with hypermethylation [3], suggesting decreased expression of the OXT-R in at least a subgroup of individuals with ASD. Abnormalities in oxytocin blood levels have also been reported in this population [6-8].

Oxytocin is a nine-amino-acid peptide, which is synthesized in the paraventricular and supraoptic nucleus of the hypothalamus and released into the bloodstream by the posterior pituitary. Oxytocin is also widely distributed in the central nervous system. Oxytocin has been shown to play a role in social recognition, memory, and attachment, as well as in stereotyped behaviors such as exaggerated grooming [9,10]. Oxytocin knockout mice exhibit deficits in social recognition, in the context of intact olfaction and cognitive abilities, which were recovered by intraventricular oxytocin [11]. OXT-R knockout mice produce fewer ultrasonic vocalizations in response to social isolation than the wild type and exhibit more aggressive behavior [12]. Sala and colleagues recently published research on phenotypic characterization of the OXT-R null mice [13]. They reported that OXT-R null mice demonstrated resistance to change and susceptibility to seizures in addition to the social deficits previously described. Of particular interest, administration of oxytocin restored to normal the social exploration and recognition deficits.

### Human studies of neuropeptide hormones

Methodological difficulties have limited the number of studies examining the effect of oxytocin on social cognition and function in typically developing humans. Early studies showed that intranasal oxytocin (IN-OXT) promotes trust and prosocial behavior in humans [14]. In a functional magnetic resonance imaging study, participants received IN-OXT or placebo and viewed fear-inducing, social (angry and fearful faces) and non-social (threatening scenes) stimuli [15]. Participants receiving oxytocin showed reduced amygdala activation to social stimuli more than nonsocial kinds of stimuli, further suggesting that oxytocin is mediating human social behavior. These findings were then replicated by Domes and colleagues, who showed that oxytocin attenuates amygdala response to faces regardless of valence [16]. Of relevance to autism, Domes and colleagues found that oxytocin facilitated performance on measures related to the ability to infer the mental states of others or that assessed aspects of theory of mind [17]. Another study found that IN-OXT increases gaze to the eye region of human faces [18], a finding or particular interest to autism, since affected individuals often have eye contact deficits. Improved face identity recognition memory was reported post 20 IU of IN-OXT administration versus placebo [19]. Recognition memory was improved for faces but not objects with IN-OXT versus placebo in a task where volunteers had to assess previously viewed faces to be known or unknown, suggesting a selective effect of oxytocin on social memory [20]. The last two studies documented social cognitive effects of a single dose of IN-OXT at 24 hours post administration, a finding suggestive of social learning effects.

### Single-dose intranasal oxytocin studies in ASD

In a double-blind, crossover challenge of intravenous oxytocin versus placebo, intravenous administration of oxytocin facilitated social learning in patients with ASD [21]. In 2003, the same authors also reported that ASD patients showed a significant reduction in repetitive behavior following oxytocin versus placebo infusion [22]. Another study randomized 16 adolescents to a crossover placebo-controlled study of a single dose of IN-OXT (24 IU for 15 to 19 year olds, 18 IU for 12 to 15 year olds) [23]. The authors showed significant improvements in the Reading the Mind in the Eyes Task (RMET) with minimal side effects. Andari and colleagues randomized 13 adults with ASD to a single dose of IN-OXT and reported that patients exhibited stronger interactions with the most socially cooperative partner during a ball game, and reported enhanced feelings of trust [6].

Given that social interaction deficits and repetitive behaviors are core symptom domains of autism, and that oxytocin is involved in the regulation of social communication and repetitive behaviors, oxytocin may present a therapeutic target for individuals with ASD. The primary aim of this study was to investigate the safety and potential therapeutic effects of IN-OXT (Syntocinon; NOVARTIS, Basel, Switzerland) versus placebo in adults with ASD, with respect to the two core symptom domains of social cognition/functioning and repetitive behaviors.

## Methods

This was a randomized, double-blind, placebo-controlled, parallel design trial of IN-OXT versus placebo in adults with ASD.

### Participants

Participants were recruited through advertisements in local media. Diagnosis was established using a diagnostic interview to establish DSM-IV criteria for an ASD supported by the Autism Diagnostic Observation Schedule [24] and the Autism Diagnostic Interview – Revised [25] performed by research-reliable administrators. All eligibility assessments were completely before randomization into the study. Other inclusion criteria included male or female outpatients 18 to 60 years of age who had a Clinician Global Impression (CGI) – severity score ≥4 (moderately ill), were on stable pharmacologic and nonpharmacologic treatments for at least 3 months, had a normal physical examination, and with full-scale IQ >70. Exclusion criteria included prematurity; primary axis I disorders such as bipolar disorder, psychosis, post-traumatic stress disorder, schizophrenia, or major depressive disorder/anxiety disorder; history of significant neurological disease, including, but not limited to, unstable epilepsy disorder, known genetic syndromes, or known abnormal brain magnetic resonance imaging (structural brain lesions); or evidence or history of malignancy or any significant hematological, endocrine, cardiovascular (including any rhythm disorder), respiratory, renal, hepatic, or gastrointestinal disease. Sexually active women had to be on two barrier methods of contraception and no hormonal birth control. Patients unable to tolerate venipuncture procedures were also excluded.

Participants were assessed for capacity to sign consent by an independent psychiatrist. Only volunteers with documented capacity in this fashion were invited to participate in the study. These participants signed the informed consent approved by the Mount Sinai Institutional Review Board and according to the Helsinki agreement. Participants underwent a comprehensive medical evaluation, baseline assessments and were randomized by the pharmacy in a one-to-one fashion. All efficacy assessments were carried out by an independent evaluator who was blinded to both side effects and group assignment. All safety evaluations were performed by a physician who was blinded to group assignment. Although we are not aware of any characteristic side effects of IN-OXT, this approach assured that all efficacy assessments were done by a truly blinded clinician.

### Medications

Oxytocin (Syntocinon; NOVARTIS) and placebo were administered in the form of intranasal spray. Participants received twice-daily 6 weeks of either 24 IU (six puffs) oxytocin or placebo, in the morning and early afternoon. Participants were instructed to sit upright, and take one puff every 30 seconds, alternating nostrils. All participants took their first dose in front of the study clinician to assure correct administration and tolerability. Placebo was normal saline in identical bottles and labels. Participants were asked to comment on smell and taste after the first dose. Only one commented on possible smell and he was randomized to the placebo. A computer-generated randomization table was created by the research pharmacist and used to randomize participants.

### Efficacy and safety assessments

Participants were seen every 2 weeks for CGI – improvement ratings, vital signs and adverse event monitoring. At each visit participants were assessed using measures of repetitive behaviors (the Yale Brown Obsessive Compulsive Scale (YBOCS) – compulsion subscale [26]) and the Repetitive Behavior Scale – Revised (RBS-R) [27]), the first being a well-established outcome measure for repetitive behaviors and the second being a dimensional, validated measure of repetitive behaviors in ASD. In the absence of well-validated outcome measures for social function in this population and this age group, we used the Social Responsiveness Scale [28]. Blood work for safety included blood count, electrolytes, liver and renal function, and osmolality. Safety bloodwork, an electrocardiogram and weight measurements were done at baseline and week 6 visits. At baseline and week 6, participants were administered social cognition/perception measures that have been shown to change in other populations or after single-dose oxytocin: the RMET – Revised [29], and the Diagnostic Analysis of Nonverbal Accuracy (DANVA-2) [30]. Quality of life was measured using the World Health Organization Quality of Life Questionnaire (WHOQOL) – emotional/social subscales [31]. Primary outcome measures included the CGI – improvement, the DANVA paralanguage test and the RBS-R. The rest of the measures were considered secondary.

The participants used a medication diary to mark down every time they took the medication. The diary was reviewed at every visit and the study clinician and the participant problem-solved together in the case of missed doses to improve compliance.

### Statistical approach

We evaluated all distributions to assess need for data transformations. We did not find any strong statistical or visual evidence of distributional concerns and so no transformations were undertaken. We then used full-information-maximum likelihood mixed-effects regression models to test the hypothesis of differential change between the treatment groups across time (time × group interaction), for all measures except for CGI – improvement. This approach conforms to intent-to-treat principles as all randomized subjects are included in the analysis. Further, for comparisons reaching statistical significance we repeated the analysis using baseline as a covariate. The CGI – improvement scales are based on clinically assessed change (improvement). We therefore used the week 6 Improvement Ratings to categorize patients as clinically improved (CGI ≤2) or not (CGI >2). Sixteen of the 19 patients (84%) had data at week 6. For the remaining three subjects, we imputed week 6 ratings using expectation-maximization (EM) methods and the earlier CGI ratings. In all three cases the imputed ratings were >2 and the patients were classified as not improved.

For the RBS-R we calculated two scores (higher-order vs. lower-order repetitive behaviors) in an effort to decrease the number of variables analyzed. This is based on previous factor analysis of the RBS-R that produced these two factors: higher order (ritualistic, sameness, compulsive and restricted subscales) and lower order (stereotypy and self-injury) [32]. This construct has also been supported by a factor analysis of the YBOCS in autism [33]. Cohen’s *d* effect sizes were calculated on observed scores to provide a sense of size of effect to guide future work. Given that this is a pilot signal finding study, no corrections for multiple comparisons were done.

## Results

**Figure 1:**
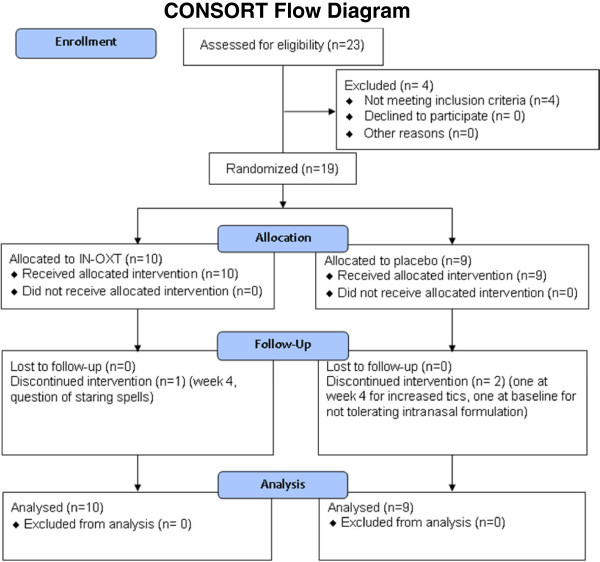
Consort flow diagram.

Nineteen adults (16 males and three females, mean age 33.2 ± 13.3 years) (Figure 1) with a diagnosis of high-functioning autism or Asperger’s disorder (Autism Diagnostic Observation Schedule –social + communication, 8.17 (3.24); Autism Diagnostic Interview – social, 18.3 (6.3); Autism Diagnostic Interview – communication, 14.2 (6.5); Autism Diagnostic Interview – repetitive, 6 (4.1)) were recruited into a 6-week randomized placebo controlled trial. Ten participants received oxytocin and nine received placebo. There were no significant differences in age (mean (standard deviation): oxytocin, 33.8 (12.7); placebo, 32.9 (14.4); *t* = 0.14, df = 17, *P* = 0.88) or full-scale IQ (mean (standard deviation): oxytocin, 99 (22); placebo, 118 (19); *t* = 1.94216, df = 16, *P* = 0.07) (Table 1).

**Table 1:** Demographic information

	**Total**	**Oxytocin**	**Placebo**
Gender			
Male	16 (84%)	9	7
Female	3 (16%)	1	2
Race			
Caucasian	14 (73.7%)	7	7
Black	1 (5.2%)	1	0
Hispanic	1 (5.2%)	1	0
Asian	2 (10.5%)	1	1
Other	1 (5.2%)	0	1
Group assignment		10 (52%)	9 (48%)
Age (standard deviation)	33.2 (13.3)	33.8 (12.7)	32.9 (14.4)
IQ (standard deviation)	107 (24)	99 (22)	118 (19)

Data presented as *n* (%) unless indicated otherwise.

A full-information maximum-likelihood mixed-effects regression analysis revealed that participants receiving oxytocin performed better on several outcome measures than the placebo group after the 6 weeks of treatment.

### Primary outcome measures

There were significant differences in performance on the RBS-R lower-order behaviors (*t* = −2.17, df = 16.78, *p* = 0.045, Cohen’s *d* effect size = 0.64) but not on the DANVA or CGI. Although baseline differences were not statistically significant, they were somewhat large and as such the analysis was repeated with baseline added as a covariate. The difference in the RBS-R lower-order behaviors was now a trend (*t* = −1.971, df = 17.18, *p* = 0.065). For the rest of the measures, co-varying baseline did not change the conclusions of the analyses. Thirty percent (3/10) of participants in the oxytocin group were rated as improved on the CGI – improvement, whereas 11% (1/9) of participants in the placebo group were rated as improved.

### Secondary outcome measures

In the analysis of secondary measures, significant improvements were noted on the RMET – Revised (*t* = 3.91, df = 9.01, p = 0.004, Cohen’s *d* effect size = 1.2) and the WHOQOL – emotional (*t* = 2.42, df = 10.89, p = 0.034, Cohen’s *d* effect size = 0.84) (Table 2). The results remained significant for the RMET (*t* = 4.045, df = 10, p = 0.002) and the WHOQOL – emotional (*t* = 2.43, df = 12.37, p = 0.031). There were no significant differences between treatment groups across the 6 weeks seen on the SRS, YBOCS, and RBS-R higher-order behaviors, and co-varying baseline did not change the conclusions of the analyses. However, this is a pilot study and therefore was not powered to detect anything but very large effect sizes. We are thus reporting all effect sizes to assist with developing future studies (Table 2).

**Table 2:** **Parameter estimates and *P *values from full-information maximum-likelihood mixed-effects regression analyses**

	***B***	**6-week *B***	***p *value**	**Mean (standard deviation)**		***d *value**
				**Week 0**	**Week 6**	
**Social cognition**						
*Primary*						
DANVA – face	2.33	13.98	0.381			0.33
Oxytocin				32.6 (8.8)	33.5 (6.4)	
Placebo				39.1 (8.4)	37.4 (7.4)	
DANVA – paralanguage	1.96	11.76	0.351			0.38
Oxytocin				27.8 (5.7)	30.5 (2.7)	
Placebo				34.1 (3.0)	35.2 (4.7)	
*Secondary*						
RMET	–	22%	**0.004**			1.2
Oxytocin			0.002*	48% (20%)	61% (24%)	
Placebo				74% (14%)	63% (12%)	
**Social function**						
*Primary*						
CGI – improvement – social			0.582			OR = 3.4
Oxytocin					30% improved	
Placebo					11% improved	
*Secondary*						
SRS	0.58	3.48	0.664			0.34
Oxytocin				92.3 (29.9)	111.4 (13.5)	
Placebo				84.5 (23.3)	96.5 (13.0)	
**Repetitive behaviors**						
*Primary*						
RBS-R higher order	–	4.57	0.301			−0.22
Oxytocin				17.0 (10.6)	17.7 (16.2)	
Placebo				20.0 (10.8)	17.8 (13.3)	
RBS-R lower order	–	−2.25	**0**.**045**			0.64
Oxytocin			0.065*	5.8 (4.6)	2.4 (2.3)	
Placebo				4.9 (3.7)	3.7 (2.6)	
*Secondary*						
YBOCS	0.16	0.96	0.220			0.13
Oxytocin				12.0 (3.9)	9.4 (2.9)	
Placebo				10.3 (2.5)	8.1 (2.5)	
**Quality of life**						
WOQOL – emotional	–	9.5%	**0.034**			0.84
Oxytocin			0.031*	47.8% (16.3%)	59.5% (16.0%)	
Placebo				65.2% (12.3%)	63.2% (12.3%)	

Means, standard deviations and standardized effect sizes are based on the observed data. *B*, parameter estimate for the group × treatment interaction showing the additional increase (or decrease) in the units of the measure for the oxytocin group per week; 6-week *B*, parameter estimate for the interaction multiplied by 6 to reflect the total differential change of the oxytocin group compared with the placebo group across the 6 weeks of the trial; *d*, Cohen’s *d* calculated from observed scores; pooled standard deviation was used (ClinTools software). For the Reading the Mind in the Eyes Task (RMET), Repetitive Behavior Scale – Revised (RBS-R) and World Health Organization Quality of Life (WOQOL) the measures were obtained pre and post, thus the parameter estimate reflects only the 6-week (pre–post) change. CGI, Clinical Global Impression; DANVA, Diagnostic Analysis of Nonverbal Accuracy; OR, odds ratio; SRS, Social Responsiveness Scale WHOQOL; YBOCS, Yale Brown Obsessive Compulsive Scale. *p values when baseline scores are included as a covariate. Significant results are in bold.

Oxytocin was well tolerated and no serious adverse effects were reported (Table 3). In the oxytocin group, two people reported increased irritability of mild to moderate severity, and two participants reported increased allergy symptoms. One participant with stable epilepsy was noted to have staring spells by his wife. The events were not observed by a physician or at any visit, but the participant was terminated for caution at week 4. Mild fatigue, a headache leg shaking and increased energy were also reported. In the placebo group, there were reports of cough, depressed mood and fatigue, all mild. There were two early terminations due to worsening tics (week 4) and a panic attack in response to use of the nasal spray (baseline visit). There were no significant or clinically important differences between oxytocin and placebo in any of the blood work, including complete blood count, electrolytes, liver/renal function and osmolality, and no abnormal electrocardiograms were reported.

**Table 3:** Safety of intranasal oxytocin in the pilot clinical trial

**Intranasal oxytocin**	**Placebo**
Irritability	Fatigue
Irritability (moderate)	Depressed mood
Nasal congestion/allergy symptoms (two individuals)	Cough
Query absence seizures	Worsening tics
Fatigue	Panic attack – adverse reaction to sensation of first intranasal spray administration (moderate)
Headache	
Leg shaking	
Increased energy	

Each cell represents one side effect in one participant, unless otherwise specified. In addition, all side effects were mild unless otherwise specified.

## Discussion

To our knowledge, this is the first study to employ an acute treatment trial of daily administration of IN-OXT in ASD. Although no significant improvements were noted in the primary outcome measures when controlling for baseline differences, 6-week use of IN-OXT versus placebo resulted in improvements in aspects of social cognition, repetitive behaviors and emotional well-being in some, although not all, measures used – suggesting potential therapeutic benefit for IN-OXT in this population, which needs to be explored in follow-up larger studies. Further, our study did not report any serious adverse effects and IN-OXT was well tolerated. Our data are consistent with previous single-dose studies in individuals with ASD. Our data are also consistent with recent data from schizophrenia and social anxiety. Feifel and colleagues published an augmentation study of oxytocin in schizophrenia [34]. In 19 participants with residual symptoms treated with 40 IU oxytocin versus placebo, the authors reported that oxytocin significantly reduced scores on the Negative Symptom Scale in addition to the Positive Symptom Scale and CGI – improvement scale compared with placebo at the 3-week end point. Oxytocin was well tolerated and no adverse effects were reported. In a randomized, double-blind, placebo-controlled trial, Guastella and colleagues administered 24 IU oxytocin or a placebo in combination with exposure therapy to 25 participants who met primary diagnosis for social anxiety disorder [35]. Participants administered with oxytocin showed improved positive evaluations of appearance and speech performance, suggesting that following exposure therapy the administration of oxytocin improved the mental representations of self.

In this study the documented effects are on social perception (RMET), lower-order repetitive behaviors (RBS-R) and quality of life as it relates to emotion (WHOQOL – emotion, patients described an effect of well-being). There is large debate in the literature about the nature of central/peripheral effects of oxytocin on cognition/perception and behavior. Both amygdala and the nucleus accumbens are rich in OXT-R. The question remains whether the effects of oxytocin on social function are related to anxiety, social reward, social perception (for example, emotion, identity detection) or social cognition (for example, theory of mind). Evidence exists for each of these hypotheses. The anxiolytic properties of oxytocin have been well documented for both exogenous and endogenous release and are mediated by both central and peripheral mechanisms [36]. However, effects on trust, theory of mind, emotion detection and positive and negative symptoms of schizophrenia have been repeatedly documented. In addition, in studies where the investigators controlled for anxiety using self-report measures (for example [16]), oxytocin doses that were adequate to produce social cognition effects did not show effects of anxiety. Still, given that the relations between anxiety, affiliative behaviors and social cognition/perception are not yet well delineated, this remains an area of active research.

The effects on repetitive behaviors were interesting, with no effects observed for higher-order behaviors (for example, compulsive-like) but improvements noted for low order behaviors (for example, stereotypy) [32,33], whereas no effects were noted in a measure that combined both types of behaviors in a single severity score (YBOCS). The possibility that the lower-order behavior score may be preferentially capturing pleasure-seeking repetitive behaviors and differentially responding to oxytocin compared with the higher-order domain, traditionally thought to be similar to the egodystonic behaviors of obsessive–compulsive disorder, is intriguing and needs to be examined further. This may be of particular interest given the paucity of available interventions for lower-order repetitive behaviors.

### Limitations

The effect sizes in our study for social cognition and function ranged from small/medium to very large. This was a short-duration (6 weeks), small-sample study and all such effect sizes need to be viewed with caution.

We acknowledge that our relatively small sample size resulted in baseline differences, which although not significant were large in some of the measures. However, the effects remained significant for both social cognition as measured by the RMET and the WHOQOL – emotional subscale after adding baseline scores as a covariate and there was a strong trend noted for improvements in repetitive behaviors as measured by the lower-order subscale of the RBS-R. In addition, the duration of the study was only 6 weeks and as such we may have underestimated the potential impact of oxytocin on core symptom domains. Furthermore, the sample size precludes us from examining the impact of other characteristics such as IQ and concurrent medications on treatment response and safety (Additional file 1). The three female participants were all premenopausal and on no hormonal contraception, but we did not collect data on the time of menstrual cycle that may potentially interact with oxytocin [37]. In addition, no information on relationship status was collected in this study.

A concern raised about trials of IN-OXT is related to the very short half-life of IN-OXT in the blood. However, in the context of documented behavioral effects lasting several hours to days, it seems that plasma levels of oxytocin are not related to such effects. Although much clarity is needed about the mechanism of the behavioral effects noted with IN-OXT administration, it is worth noting that levels of vasopressin in the cerebral spinal fluid were still rising 4 hours post administration of a single intranasal dose of the peptide in a cerebral spinal fluid pharmacokinetic study [38].

Placebo in this study was normal saline. Although the participants detected no taste/smell for the active compound, normal saline is not the vehicle for intranasal Syntocinon and future studies should use placebo with identical inactive ingredients.

Lastly, the study did not follow-up the participants once the medication had been stopped. Given the animal literature suggesting that oxytocin promotes neural plasticity changes, we would predict that any effects should outlast the period of actual administration. Follow-up studies need to include such a design.

## Conclusions

Our pilot study supports the therapeutic potential and safety of daily administration of IN-OXT for social cognition/function deficits and possibly repetitive behaviors in adults with ASD. Larger sample studies of longer duration are required to fully examine these effects.

## Abbreviations

ASD: Autism spectrum disorder; CGI: Clinical Global Impression; DANVA: Diagnostic Analysis of Nonverbal Accuracy; Df: Degrees of freedom; IN-OXT: Intranasal oxytocin; OXT-R: Oxytocin receptor; RBS-R: Repetitive Behavior Scale – Revised; RMET: Reading the Mind in the Eyes Task; SRS: Social Responsiveness Scale; WHOQOL: World Health Organization Quality of Life; YBOCS: Yale Brown Obsessive Compulsive Scale.

## Competing interests

EA has consulted without fees to Proximagen, and Neuropharm and has received a consultation fee from Seaside Therapeutics and NOVARTIS. EH has applied for a patent for oxytocin in autism. This was true during the course of this study. As such, this study was reviewed by the Conflict of Interest Committee of Mount Sinai School of Medicine and the principal investigator (EA) confirms that it was run as per the management plan developed by this committee, which included subcontracting data analysis to WC at St John’s University, and Data Safety Monitoring Board oversight . EH has consulted in the past for Neuropharm and Nastech. The remaining authors declare that they have no competing interests.

## Authors’ contributions

EA was the principal investigator for the study; she designed and ran the study, and prepared the manuscript. LS contributed to designing the study, supervised psychological assessments, and contributed to manuscript preparation. WC performed the data analyses and contributed to manuscript preparation. JB contributed to conceptualization and design of the study as well as manuscript preparation. DH, SW, ATW, and LP contributed to data acquisition and manuscript preparation. NT and AK contributed to interpretation of the data and manuscript preparation. EH was critical to conceptualization of the study and contributed to manuscript preparation. All authors read and approved the final manuscript.

## Supplementary Material

Additional file 1 **Concomitant psychotropic medications.** Table S1 presents all concomitant medications taken by participants during the study. Click here for file
