# Supplementary material for: Intranasal oxytocin versus placebo in the treatment of adults with autism spectrum disorders: a randomized controlled trial
Source: Mol Autism. 2012 Dec 5;3:16. doi: 10.1186/2040-2392-3-16 (PMC3539865; doi:10.1186/2040-2392-3-16)
Supplement: Additional file 1 — Concomitant psychotropic medications. Table S1 presents all concomitant medications taken by participants during the study. [file 2040-2392-3-16-S1.docx]

Table S1 : Concomitant psychotropic medications

|  | Oxytocin (n) | Placebo (n) |
| --- | --- | --- |
| primidone* | 1 |  |
| divalproex sodium** | 2 |  |
| risperidone | 1 |  |
| aripiprazole |  | 1 |
| fluoxetine |  | 1 |
| wellbutrin | 1 |  |
| sertraline |  | 1 |
| duloxetine |  | 1 |
| alprazolam | 1 |  |
| Amphetamine/dextroamphetamine | 1 |  |
| atomoxetine |  | 1 |

* for seizure control

** for seizure control in one participant and for mood lability in another participant
